# Characterization of a *mcr-1* and CRISPR-Cas System Co-harboring Plasmid in a Carbapenemase-Producing High-Risk ST11 *Klebsiella pneumoniae* Strain

**DOI:** 10.3389/fmicb.2021.762947

**Published:** 2021-10-27

**Authors:** Yi-Hsiang Cheng, Sheng-Hua Chou, Po-Han Huang, Tsuey-Ching Yang, Yu-Fan Juan, Barry N. Kreiswirth, Yi-Tsung Lin, Liang Chen

**Affiliations:** ^1^Division of Infectious Diseases, Department of Medicine, Taipei Veterans General Hospital, Taipei, Taiwan; ^2^Institute of Emergency and Critical Care Medicine, National Yang Ming Chiao Tung University, Taipei, Taiwan; ^3^Department of Biotechnology and Laboratory Science in Medicine, National Yang Ming Chiao Tung University, Taipei, Taiwan; ^4^Division of Microbiology, Department of Pathology and Laboratory Medicine, Taipei Veterans General Hospital, Taipei, Taiwan; ^5^Hackensack Meridian Health Center for Discovery and Innovation, Nutley, NJ, United States; ^6^Department of Medical Sciences, Hackensack Meridian School of Medicine, Nutley, NJ, United States

**Keywords:** colistin, carbapenemase, fitness cost, high-risk clone, *mcr-1*

## Abstract

We set out to study the prevalence of the *mcr-1* gene in carbapenemase-producing *Klebsiella pneumoniae* (CPKP) strains, and to determine whether its presence is associated with a fitness cost. A total of 234 clinical CPKP isolates were collected from a tertiary medical center in Taiwan from January 2018 to January 2019. The *mcr-1* and carbapenemase genes were detected by polymerase chain reaction (PCR) followed by Sanger sequencing. The *mcr-1*-positive carbapenemase-producing strain was characterized by whole genome sequencing, a plasmid stability test and a conjugation assay. *In vitro* growth rate and an *in vivo* virulence test were compared between the parental *mcr-1*-positive strain and its *mcr-1* plasmid-cured strain. We identified only one *mcr-1* positive strain (KP2509), co-harboring *bla*_*KPC–*__2_ and *bla*_*OXA–*__48_, among 234 (1/234, 0.43%) CPKP strains. KP2509 and its *Escherichia coli mcr-1* transconjugant showed moderate colistin resistance (MIC = 8 mg/L). The *mcr-1* is located on a large conjugative plasmid (317 kb), pKP2509-MCR, with three replicons, IncHI, IncFIB, and IncN. Interestingly, a complete Type IV-A3 CRISPR-Cas system was identified in pKP2509-MCR. Plasmid pKP2509-MCR was highly stable in KP2509 after 270 generation of passage, and the pKP2509-MCR cured strain PC-KP2509 showed similar growth rate and *in vivo* virulence in comparison to KP2509. The prevalence of *mcr-1* in CPKP strains remains low in our center. Notably, we identified a large plasmid with multiple replicons containing both the *mcr-1* and the Type IV-3A CRISPR-Cas genes. The further spread of this highly stable plasmid raises concern that it may promote the increase of *mcr-1* prevalence in CPKP.

## Introduction

The worldwide spread of carbapenemase-producing *Klebsiella pneumoniae* (CPKP) poses a significant threat to public health (Navon-Venezia et al., 2017; Bonomo et al., 2018), and colistin is one of the last-resort drugs to treat CPKP infections (Poirel et al., 2017). Of clinical concern, the occurrence of colistin resistance in CPKP has been increasingly reported in clinical cases worldwide, and were commonly associated with high mortality (Poirel et al., 2017). The major mechanism of colistin resistance involves the modification of lipid A with cationic groups, such as 4-amino-4-deoxy-L-arabinose or phosphoethanolamine (pEtN) (Baron et al., 2016). Modifications of lipid A are usually mediated by chromosomal mutations of genes encoding the two component systems PmrAB, PhoPQ, and CrrAB or inactivation of the *mgrB* gene, a negative regulator of the PhoP/PhoQ system (Baron et al., 2016). Notably, the plasmid-mediated mobile colistin resistance gene *mcr-1*, was first described in *Escherichia coli* and *K. pneumoniae* strains isolated in China between 2011 and 2014 (Liu et al., 2016). The encoded MCR protein is a pEtN transferase that is able to modify the negatively charged phosphate groups of lipid A with positively charged pEtN residues resulting in colistin resistance (Liu et al., 2016).

*mcr-1* has been reported in various genera of the *Enterobacterales* isolated from environmental, animal, and human samples (Liu et al., 2016). *E. coli* remains as the most prevalent species carrying the *mcr-1* gene, while a lower prevalence is observed in *K. pneumoniae* (Poirel et al., 2017; Quan et al., 2017). It has been hypothesized and commonly proposed that the development of antibiotic resistance often carries a cost to biological fitness (Andersson and Levin, 1999). Previous studies showed the presence of a recombinant *mcr-1*-bearing plasmid resulted in a growth defect in *K. pneumoniae* (Nang et al., 2018; Tietgen et al., 2018), but it did not impair fitness in *E. coli*. This finding partially explains why *mcr-1* is more frequently detected in *E. coli* and less abundant in *K. pneumoniae* (Wang et al., 2017). The emergence of *mcr-1* genes in CPKP is particularly concerning even though only sporadic clinical CPKP isolates with *mcr-1* have been reported (Di Pilato et al., 2016; Aires et al., 2017; Berglund et al., 2018; Dalmolin et al., 2018; Mendes et al., 2018; Srijan et al., 2018; Giordano et al., 2019; Wang et al., 2021). Studies regarding the impact of *mcr-1* on the fitness of CPKP remain to be evaluated.

In this study, we examined the prevalence of *mcr-1* in clinical CPKP isolates collected from a large tertiary hospital in Taiwan. We then investigated the genomic characteristics of the CPKP strain carrying *mcr-1* by whole genome sequencing, and explored the potential fitness cost attributable to *mcr-1* in CPKP.

## Materials and Methods

### Microbiological Characterization of Carbapenemase-Producing *Klebsiella pneumoniae* Strains

Carbapenemase-producing *Klebsiella pneumoniae* strains isolated from clinical specimens in Taipei Veterans General Hospital (TVGH) during January 2018 to January 2019 were consecutively collected in this study. Only the first culture was included in this study, regardless of the number of positive CPKP isolates that were recovered from the patient. The study protocol was approved by the Institutional Review Board at TVGH. The informed consent form was waived. Bacterial identification and antimicrobial susceptibility testing of CPKP were shown in Supplementary Methods. Genes encoding for carbapenemases (*bla*_*KPC*_, *bla*_*NDM*_, *bla*_*IMP*_, and *bla*_*OXA–*__48_) and for *mcr-1* were determined by polymerase chain reaction (PCR) as previous described (Quan et al., 2017; Huang et al., 2018). S1-nuclease pulsed-field gel electrophoresis (S1-PFGE) for plasmid profiling was performed as previously described (Barton et al., 1995). The whole genome sequencing of the *mcr-1*-harboring CPKP (KP2509) and comparative genomics were detailed in Supplementary Methods.

### Transferability and Stability of *mcr-1*-Encoding Plasmid

The conjugation assay followed the method described in previous work with minor modifications (Huang et al., 2018). KP2509 was the donor strain and *E. coli* J53 (sodium azide resistant) was the recipient strain. In brief, both donor strain and recipient strain were mixed with about 1 × 10^8^ cfu and dotted on sterilized filter paper, which was then incubated on an LB agar plate for 18 h at 37°C. We used LB agar plates supplemented with 2 mg/L colistin and 100 mg/L sodium azide to select transconjugants. PCR for *mcr-1* was performed to confirm the transconjugants and the MICs of colistin were determined.

We then evaluated the stability of *mcr-1*-harboring plasmid. In brief, bacterial cultures were grown at 37°C in a shaking incubator (200 rpm) and daily passaged using a 1:1000 dilution in antibiotic-free LB broth for 27 days. One hundred colonies were randomly selected from the plated culture, and replica and inoculated onto colistin (2 mg/L) containing and an antibiotic-free LB agar plate daily. The colonies growing on antibiotic-free LB agar plates but not on colistin-containing LB agar plates were further examined for the presence of *mcr-1* by PCR. The resulting *mcr-1* plasmid-cured strain, named PC-KP2509, was involved in the following experiments.

### Construction of *mcr-1*-Bearing Recombinant Plasmid and Evaluation of Its Stability

The *mcr-1*-bearing recombinant plasmid with the tetracycline resistant gene (*tetA*) designated as pJET-TC-MCR-1 (7,489 bps) was generated. We then transformed the pJET-TC-MCR-1 and pJET-TC (control) plasmids into the *mcr-1* plasmid-cured strain PC-KP2509, and compared their stabilities in the common host. The transformants were passaged daily without additional antibiotics for 28 days. In addition, we compared the relative copy numbers between pJET-TC-MCR-1-bearing PC-KP2509 and KP2509 by real-time qPCR experiments. The details were shown in Supplementary Methods.

### Evaluation of *in vitro* Growth Curve

To assess the impact of having the *mcr-1* plasmid on bacterial fitness, we conducted the growth curve analysis for both KP2509 and PC-KP2509. Briefly, bacteria were planktonically cultured at 37°C, adjusted to the same OD_600_ of 0.1, and inoculated into antibiotic-free LB broth and incubated for 6 h, with OD_600_ measurements at 0.5, 1, 1.5, 2, 2.5, 3, 4 and 6 h by spectrometer, and the statistical difference was analyzed by Mann–Whitney U test (GraphPad).

### Experiments to Assess *in vivo* Fitness Using a Mouse Lethality Model

KP2509 and PC-KP2509 strains were compared in a mouse lethality study to determine the 50% lethal dose (LD50). Female 6–8-week-old C57BL/6 mice were administered with an intraperitoneal injection of KP2509 or PC-KP2509 strains at various concentrations of inoculum, as previously described (Huang et al., 2018). Five mice were used for each concentration of inoculum. The survival rate was determined by Kaplan–Meier analysis with a log-rank test, using the Prism software package (GraphPad). All animal care procedures and protocols were approved by the institutional animal care and use committee at the National Yang Ming Chiao Tung University.

## Results

### Microbiological Characteristics of the Carbapenemase-Producing *Klebsiella pneumoniae* With a *mcr-1*-Bearing Plasmid

During the study period, a total of 195 KPC- 2-, 32 OXA-48-like- (OXA-48, *n* = 31; OXA-181, *n* = 1), 9 NDM- (NDM-1, *n* = 1; NDM-4, *n* = 5; NDM-5, *n* = 3), and one IMP-19-producing CPKP strains were identified, including three strains carrying both *bla*_*KPC–*__2_ and *bla*_*OXA–*__48_. However, only one *mcr-1*-positive CPKP strain (KP2509) was identified, and interestingly, this strain simultaneously carried *bla*_*KPC–*__2_ and *bla*_*OXA–*__48_. Strain KP2509 was isolated from a 79-year-old male who died of bacteremia. Two months before this episode of bacteremia, the patient experienced a sudden cardiac arrest and gained vital signs after cardiopulmonary resuscitation. He was placed on a mechanical ventilator and suffered from repeated episodes of pneumonia and received prolonged broad-spectrum antibiotics, including cefepime, vancomycin, cefoperazone/sulbactam, levofloxacin, and colistin. This patient was later treated with tigecycline plus colistin, but his health condition continued to deteriorate, and died 3 weeks after the bacteremia. Strain KP2509 was only susceptible to tigecycline, amikacin, and ceftazidime/avibactam (Table 1).

**TABLE 1:** MICs (mg/L) of antibiotics in the strains.

	KP2509	PC-KP2509	*E. coli* J53	Transconjugant
Ampicillin/sulbactam	≥32	≥32	≤2	≥32
Piperacillin/tazobactam	≥128	≥128	≤4	≥128
Cefazolin	≥ 64	≥64	≤4	8
Cefuroxime	≥ 64	≥64	4	16
Ceftazidime	≥ 64	≥64	≤1	≤1
Ceftriaxone	≥ 64	≥64	≤1	≤1
Cefepime	≥ 64	≥64	≤1	≤1
Ertapenem	≥ 8	≥8	≤0.5	≤0.5
Imipenem	≥ 16	≥16	≤0.25	≤0.25
Amikacin	≤ 2	≤2	≤2	≤2
Gentamicin	≥ 16	≤1	≤1	≥16
Ciprofloxacin	≥ 4	≥4	≤0.25	≤0.25
Levofloxacin	≥ 8	≥8	≤0.12	≤0.12
Colistin	8	0.5	0.5	8
Tigecycline	2	1	0.75	1
Ceftazidime/avibactam	4	4	NA	NA

*NA, non-available.*

### Whole Genome Sequencing of Strain KP2509

The hybrid assembly using the combination of Illumina (∼ 180×) and PacBio (∼ 780×) sequencing data successfully closed the genome of K2509, resulting in one complete chromosome and five closed plasmids. KP2509 contains a 5,470,223 bp chromosome with an average G + C content of 57.4% and containing 5,292 predicted open reading frames. In addition, it harbors five plasmids ranging in size from 10 to 317 kb. *In silico* multilocus sequencing typing (MLST) revealed that KP2509 belongs to the successful ST11 clone with KL47 capsular type. The detailed analysis of acquired antimicrobial resistance was shown in Supplementary Results. In addition, KP2509 contains the yersiniabactin biosynthetic operon (*ybt, irp*, and *fyuA*), whereas the other known virulence markers for hypervirulent *K. pneumoniae* strains, such as *rmpA*/*A2* and *iucABCD*, were absent in KP2509.

### Genomic Characterization of Plasmids in KP2509

The five plasmids in KP2509 belong to IncHI-FIB-N, IncFII-R, IncL, IncN, and ColRNAI incompatibility groups. Among them, *bla*_*KPC–*__2_ is carried on plasmid pKP2509-KPC, which is 100,684 bp in length, and co-carries ESBL resistance genes *bla*_*CTX–M–*__65_ and *bla*_*SHV–*__12_, and chloramphenicol resistance gene *catA2*. Carbapenemase gene *bla*_*OXA–*__48_ was located on the IncL plasmid pKP2509-OXA, which is 63,769 bp in size. The detailed analysis of these plasmids was shown in Supplementary Results.

**FIGURE 1:**
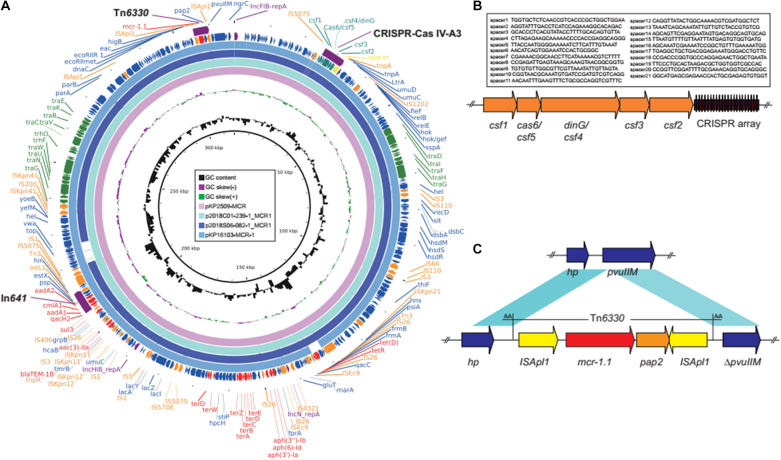
**(A)** Plasmid structure of *mcr*-1 and CRISPR-Cas co-harboring plasmids. Colored arrows indicate open reading frames, with purple, orange, green, red, teal, and blue arrows representing replication genes, mobile elements, plasmid transfer genes, the antimicrobial and heavy metal resistance gene, CRISPR-Cas, and plasmid backbone genes, respectively. **(B)** The CRISPR-Cas IV-A3 region in pKP2509-MCR. **(C)** The *mcr-1*-harboring Tn*6330* and neighboring genes in pKP2509-MCR. Blue shading denotes regions of shared homology among different elements. *hp*, hypothetical protein gene.

The colistin resistant gene *mcr-1.1*, was found on a multi-replicon (IncHIB-FIB-N) plasmid, pKP2509-MCR, which is 317,010 bp in size, and co-harbors resistance genes, *aph(3″)-Ib*, *aph(6)-Id*, *aph(3′)-Ia*, *aac(3)-IIe*, *aadA1*, *aadA2*, *bla*_*TEM–*__1_, *cmlA1*, *qacL*, and *sul3* (Figure 1A). *mcr-1* is located in an IS*Apl1*-*mcr-1*-*pap2*-IS*Apl1* composite transposon (Tn*6330*), and inserted into a DNA-methyltransferase gene with the 2-nt direct repeat (AA) (Figure 1C), indicating the plasmid acquisition of the *mcr-1* gene was through transposition. pKP2509-MCR harbors a class I integron (In*641*) with the antimicrobial resistance gene cassette: *estX3*-*psp*-*aadA2*-*cmlA1*-*aadA1a*-*qacH2*. In addition, a complete conjugation transfer operon was identified in pKP2509-MCR.

Interestingly, mining the pKP2509-MCR sequences identified a ∼6.4 kb Type IV-A3 CRISPR-Cas (clustered regularly interspersed short palindromic repeats-CRISPR associated protein) system, located upstream of the mutagenesis regulatory genes *umuD* (Figures 1A,B). The Type IV-A3 CRISPR-Cas system includes *casf1* (*cas8*-like, large subunit), *csf5* (*cas6*-like, endoribonuclease for crRNA process), *csf4* (*dinG*, structure-specific helicase), *csf3* (*cas5*-like), *csf2* (*cas7*-like), and CRISPR array. The CRISPR array contains 22 29-nt repeats and 21 25–33-nt spacers (Figure 1B). BLAST analysis of the spacer sequences against the NCBI database identified seven spacers, matched sequences on plasmids or prophage like sequences (spacer 1, 2, 5, 18, and 20 for plasmids and 15 and 19 for prophages), while no significant matches were found for other spacers. In addition, none of the spacer sequences were found to match either the chromosome or plasmid sequences in KP2509 (except for the CRISPR array region on pKP2509-MCR).

BLAST analysis of pKP2509-MCR identified several highly similar plasmids (e.g., pKP16103-MCR-1, MH733011; p2018C01-239-1_MCR1, CP044386; and p2018S06-082-1_MCR1, CP044377) of over >97% query coverage and >99.3% nucleotide identity (Figure 1A), containing the same Tn*6330* (*mcr-1* transposon) integration site, Type IV-A3 CRISPR-Cas genes and spacer sequences. All the three plasmids were from *K. pneumoniae* isolated in Taiwan.

### Mobility and Stability of the Natural *mcr-1*-Bearing Plasmid (pKP2509-MCR)

**FIGURE 2:**
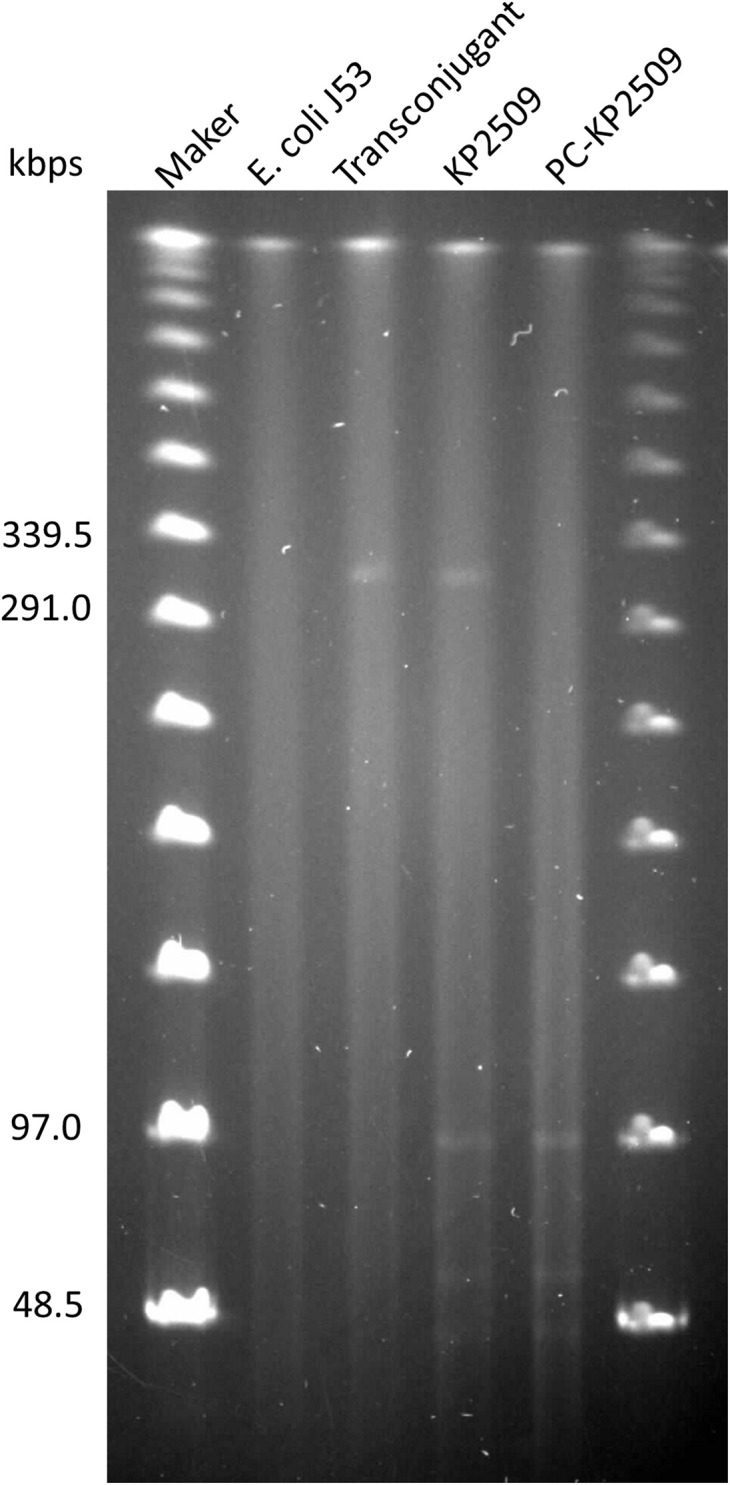
Genomes of *E. coli* J53 (lane 2), transconjugant (lane 3), KP2509 (lane 4) and PC-KP2509 (lane 5) were treated with S1-nuclease, and the resulting products were subsequently analyzed by PFGE. The profile showed an approximately 300 kbps plasmid in the transconjugant, that is consistent with the *mcr-1*-harboring plasmid from KP2509. Only four plasmids of KP2509 were detected in the gel, because the small size (10,060 bp) of the undetectable plasmid, pKP2509-5, was below the lower limit of markers.

The *mcr-1-*harboring plasmid pKP2509-MCR can be successfully transferred to *E. coli* J53 *via* solid mating, with the efficiency of 2.24 × 10^–4^ (transconjugants/recipients). The transconjugant had an increased colistin MIC of 8 mg/L (Table 1). S1-PFGE showed an approximately 300 kb plasmid in the transconjugant, which is consistent with the genomic sequencing result described above (Figure 2).

We next evaluated the stability of the *mcr-1*-harboring pKP2509-MCR in the parental KP2509 strain. After 27 days of passage (i.e., ∼ 270 generations), a single colony was isolated (PC-KP2509) among 100 replicas that was thought to be cured of the *mcr-1*-containing plasmid. The absence of *mcr-1* and the presence of *bla*_*KPC–*__2_ and *bla*_*OXA–*__48_ in PC-KP2509 strain was confirmed by PCR. PC-KP2509 showed a decreased colistin MIC of 0.5 mg/L compared to an MIC of 8.0 mg/L with the parental KP2509 (Table 1). S1-PFGE showed the absence of an approximately 300 kb plasmid in the PC-KP2509 strain compared to KP2509 (Figure 2). We repeated the same experiment to confirm the high stability of *mcr-1* bearing plasmid in KP2509 and again, we only identified one *mcr-1* plasmid cured colony after 29 days of passage.

**FIGURE 3:**
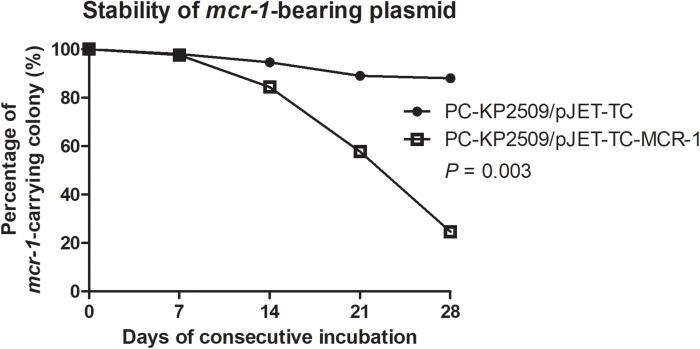
The stability of pJET-TC and pJET-TC-MCR-1 plasmids in PC-KP2509 was compared after consecutive passage. Without antibiotic pressure, pJET-TC-MCR-1 was lost from PC-KP2509 within 7 days (i.e., ∼ 70 generations), and only 24.7% PC-KP2509 population carried the *mcr-1* recombinant plasmid on Day 28. In contrast, 88% PC-KP2509 population kept pJET-TC on Day 28. The percentage of *mcr-1*-bearing colony was performed from the three independent experiments. The statistical difference was analyzed by two-way ANOVA.

Our findings showed that the large *mcr-1*-bearing plasmid pKP2509-MCR is highly stable in KP2509 strain, which appears to be inconsistent with previous reports that the expression of *mcr-1* reduced fitness in *K. pneumoniae* strains. We speculated that the high plasmid stability may be attributable to the pKP2509-MCR plasmid instead of the *mcr-1* gene, and in this case a recombinant *mcr-1*-bearing plasmid would not be as stable as the natural pKP2509-MCR plasmid. To test this hypothesis, we constructed a *mcr-1* recombinant plasmid (pJET-TC-MCR-1, 7,489 bps) and transformed it into PC-KP2509, followed by evaluating its plasmid stability. The colistin MIC of pJET-TC-MCR-1-bearing PC-KP2509 was 8 mg/L, suggesting the functional expression of *mcr-1*. Culturing the strain without antibiotic pressure, pJET-TC-MCR-1 in PC-KP2509 showed instability within 7 days (i.e., ∼ 70 generations), and only 24.7% of the PC-KP2509 population harbored the *mcr-1* recombinant plasmid on Day 28 (Figure 3). In contrast, 88% PC-KP2509 population kept the control plasmid (pJET-TC, 5,674 bps) on Day 28 (Figure 3), which is significantly higher than the frequency for pJET-TC-MCR-1 (*P* < 0.01), a result in support of the above hypothesis. Furthermore, we compared the *mcr-1* gene copy number between KP2509 and pJET-TC-MCR-1-bearing PC-KP2509 using qRT-PCR. The results showed that the copy number of *mcr-1* in pJET-TC-MCR-1-bearing PC-KP2509 was 3.9-fold higher compared to KP2509 (*P* = 0.02).

### Effects of Fitness Cost and Virulence Associated With the Carriage of pKP2509-MCR

**FIGURE 4:**
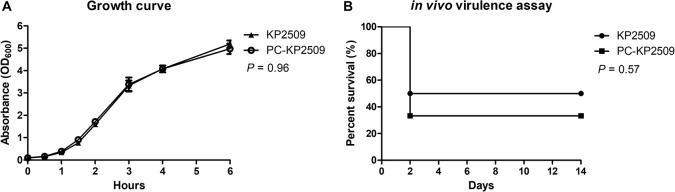
**(A)** The growth curves of KP2509 and PC-KP2509 strains were observed during 0–6 h, respectively, and the tendency of KP2509 was similar that of PC-KP2509 (*P* = 0.96). Each value was performed from the three independent experiments, and the statistical difference was analyzed by Mann–Whitney U test. **(B)** KP2509 and PC-KP2509 strains with 2.1 × 10^6^ cfu were intraperitoneal inoculated into mice, and survival rates were compared during 2 weeks. No statistical difference in Kaplan–Meier analysis with a log-rank test (*P* = 0.57).

We investigated the physiological influences associated with the plasmid pKP2509-MCR in KP2509. The growth curve of KP2509 and PC-KP2509 was highly similar (Figure 4A), suggesting pKP2509-MCR imposed limited fitness cost on the growth of the host bacteria, which is consistent with the results of plasmid stability described above. Further, the *in vivo* virulence test using a mice sepsis model showed a similar mortality rate in mice inoculated with KP2509 and PC-KP2509 at the dose of 2.1 × 10^6^ cfu (50 versus 66.7%, *P* = 0.57) (Figure 4B). We used one ST11 OXA-48-producing strain (NCRE35), with an LD_50_ of 1 × 10^7^ cfu and one hypervirulent strain (KP93) with capsular Type K1 with an LD_50_ value of 100 cfu as the control strains in the *in vivo* virulence test. Together, our results provide sound evidence that the presence of the *mcr-1*- plasmid pKP2509-MCR did not significantly compromise the *in vitro* growth and *in vivo* virulence in *K. pneumoniae* ST11 strain KP2509.

## Discussion

In this study, we only found one *mcr-1*-positive carbapenemase-producing strain (1/234, 0.4%) during a 13-month collection period. This strain belonged to the highly successful ST11 clone, and produced dual-carbapenemases, KPC-2 and OXA-48. To the best of our knowledge, this is the first report of an ST11 strain co-harboring *bla*_*KPC–*__2_, *bla*_*OXA–*__48_, and *mcr-1*, highlighting that plasmid-mediated resistance continues to reshape the antimicrobial resistome in epidemic drug-resistant strains.

The low prevalence of *mcr-1*-positive CPKP could be potentially due to the fitness cost attributed to *mcr-1*. Yang et al. (2017) demonstrated a significantly decreased growth rate, cell viability and fitness in a *mcr-1*-overexpressed *E. coli*. Similarly, Nang et al. (2018) reported that the recombinant *mcr-1*-bearing plasmid was unstable in a capsular Type K2 *K. pneumoniae* strain, and that harboring *mcr-1* contributed to reductions in *in vitro* competition studies and *in vivo* bacterial viability. However, in the current study, we compared the fitness cost of the pKP2509-MCR cured strain and its parental carbapenemase-producing strain and found that pKP2509-MCR did not interfere with the growth rate and *in vivo* virulence of KP2509. By contrast, the *mcr-1* recombinant plasmid, pJET-TC-MCR-1, was significantly less stable in comparison to the empty plasmid vector, which is consistent with previous study that *mcr-1* expression conferred a fitness cost to the bacterial host (Nang et al., 2018; Tietgen et al., 2018). Together, the results suggest that the genetic background of the large *mcr-1*-bearing IncHI-FIB-N plasmid (∼ 317 kb), pKP2509-MCR, may overcome the fitness cost imposed by *mcr-1* expression in KP2509.

Plasmids can increase their stability by conjugal transfer and/or by plasmid-encoded stability systems, including multimer resolution, plasmid partition and post-segregational killing systems (Sengupta and Austin, 2011). The pKP2509-MCR had a high *in vitro* conjugative efficiency of 2.24 × 10^–4^ (transconjugants/recipients), which may help to maintain the plasmid. In addition, the pKP2509-MCR harbors post-segregational killing *relB*/*relE* and *yoeB*/*yefM* toxin-antitoxin modules and the plasmid partitioning gene *parAB*, which may also play a role in the plasmid stability in pKP2509-MCR.

Interestingly, a novel Type IV-A3 CRISPR-Cas system was identified in plasmid pKP2509-MCR (Figure 1B). Type IV CRISPR-Cas was previously named the Unknown Type (Type U), due to its rare occurrence and its lack of adaptation modules, but was recently assigned as Type IV (Makarova et al., 2015, 2020; Newire et al., 2020). Type IV CRISPR-Cas system includes five subtypes and eight variants (A1, A2, A3, B, C, D, and E), with A3 and B being the most common (Pinilla-Redondo et al., 2020). Unlike other CRISPR-Cas systems, Type IV CRISPR–Cas modules were primarily encoded by plasmids or by prophage genomes (Makarova et al., 2015, 2020; Koonin and Makarova, 2017). Among them, Type IV-A3 system has been significantly linked to IncHI1B-FIB plasmids in *Enterobacterales* (Kamruzzaman and Iredell, 2020; Newire et al., 2020). This system contains a CRISPR array with multiple spacers primarily targeting plasmid-like elements (Pinilla-Redondo et al., 2020). However, Type IV systems lack the adaption module (*cas1* and *cas2*) and nuclease gene for target cleavage (*cas3* or *cas10*). As such, it is suggested that Type IV systems may function in a similar manner to the artificially developed catalytically dead CRISPR-Cas interference systems, which bind DNA targets but lack cleavage activity (Pinilla-Redondo et al., 2020). In this case, the Type IV systems could potentially enhance plasmid propagation and/or stabilize maintenance through interfering with host expression profiles. Currently, it remains unclear how Type IV system protect or stabilize the resident Type IV-A CRISPR-Cas carrying plasmid but a recent study in *Pseudomonas aeruginosa* has shown Type IV-A system can interfere with plasmid invasion (Crowley et al., 2019).

In addition, recent studies showed that maintaining *mcr-1* plasmid at low copy number is essential for its persistence (Yang et al., 2017, 2021). In the IncI2 plasmid, the plasmid encoded plasmid copy number repressor PcnR balances the *mcr-1* expression and bacteria fitness through repressing the plasmid copy number (Yang et al., 2021). Although the *pcnR* gene was not found in our IncHI-FIB-N plasmid pKP2509-MCR, plasmid copy number analysis showed that the recombination plasmid, pJET-TC-MCR-1, had ∼ 3.8-fold higher copy number in comparison to the pKP2509-MCR in KP2509. We suspected that the lower copy number of pKP2509-MCR may in part contribute to the plasmid stability of pKP2509-MCR in KP2509.

In this study, the *mcr-1* positive and dual carbapenemases-producing *K. pneumoniae* KP2509 was recovered from a fatal case with bacteremia, highlighting the potential threat of this emerging superbug. Notably, other *mcr-1* and type IV-A3 CRISPR-Cas system co-harboring plasmids have been isolated from different regions in Taiwan since 2018 (Chen et al., 2021), suggesting this type of *mcr-1*-carrying plasmid has already spread in Taiwan. Although the presence of *mcr-1* and carbapenemases were not frequently detected in *K. pneumoniae* (Di Pilato et al., 2016; Aires et al., 2017; Berglund et al., 2018; Dalmolin et al., 2018; Mendes et al., 2018; Srijan et al., 2018; Giordano et al., 2019; Wang et al., 2021), the further dissemination of highly stable pKP2509-MCR-like plasmids into clinical carbapenem-resistant *K. pneumoniae* could potentially change the current epidemiology, leading to the spread of colistin and carbapenem resistant successful clones.

This study was limited by the only one *mcr-1* positive CPKP strain collected, and more clinical *mcr-1* positive CPKP strains are needed to further elucidate the role of plasmid-mediated CRISPR-Cas associated with the biology of MCR-1 in CPKP. The other major limitation was that we only screen *mcr-1* in CPKP strains, thus we may miss other *mcr* genes in either CPKP or non-carbapenemase-producing carbapenem-resistant strains. Finally, was that we did not conduct the competition experiment of KP2509 and KP2509 deleting *mcr-1*. One recent study showed that the deletion of *mcr-1* in the plasmid showed a long-term competitive advantage over the parental strain in competitive co-cultures (Yang et al., 2021).

## Conclusion

In conclusion, we identified one *mcr-1*-positive strain from 234 unique CPKP strains. The *mcr-1* is harbored on a large plasmid, pKP2509-MCR, with three replicons (IncHI-FIB-N) and contains a novel Type IV-3A CRISPR-Cas system. The plasmid is highly stable in carbapenemase-producing host, without an apparent fitness cost or reduced *in vivo* virulence. The further transfer of pKP2509-MCR-like plasmid raises concern that it may increase the prevalence of *mcr-1* in CPKP, and heightens the need for surveillance of multidrug resistant *K. pneumoniae*.

## Data Availability Statement

The datasets presented in this study can be found in online repositories. The complete genome sequences of KP2509 were submitted to GenBank under the accession numbers CP065949–CP065954.

## Ethics Statement

The studies involving human participants were reviewed and approved by the Institutional Review Board at Taipei Veterans General Hospital (TVGH). Written informed consent for participation was not required for this study in accordance with the national legislation and the institutional requirements.

## Author Contributions

Y-HC did the laboratory experiment, analyzed the data, and drafted this manuscript. S-HC did the laboratory experiment. P-HH analyzed the data and drafted this manuscript. T-CY assisted the laboratory experiment. Y-FJ collected the strains and assisted the laboratory experiment. BK assisted the genomic studies. Y-TL planned and designed the study, analyzed the data, and drafted this manuscript. LC did the genomic studies and contributed to the study design. All authors have approved the final version of the article.

## Conflict of Interest

The authors declare that the research was conducted in the absence of any commercial or financial relationships that could be construed as a potential conflict of interest.

## Publisher’s Note

All claims expressed in this article are solely those of the authors and do not necessarily represent those of their affiliated organizations, or those of the publisher, the editors and the reviewers. Any product that may be evaluated in this article, or claim that may be made by its manufacturer, is not guaranteed or endorsed by the publisher.
