# Supplementary material for: Characterization of a mcr-1 and CRISPR-Cas System Co-harboring Plasmid in a Carbapenemase-Producing High-Risk ST11 Klebsiella pneumoniae Strain
Source: Front Microbiol. 2021 Oct 27;12:762947. doi: 10.3389/fmicb.2021.762947 (PMC8579119; doi:10.3389/fmicb.2021.762947)
Supplement: Supplementary file 1 [file Presentation_1.pdf]

SUPPLEMENTARY METHODS

Bacterial identification and antimicrobial susceptibility testing

The identification of *K. pneumoniae* was performed using matrix-assisted laser desorption-ionization time-of-flight mass spectrometry (bioMérieux). Antimicrobial susceptibility was determined using the Vitek 2 System (bioMérieux) except for colistin, tigecycline and ceftazidime/avibactam. The minimum inhibitory concentrations (MICs) of colistin and ceftazidime/avibactam were determined by broth microdilution method, and the MIC of tigecycline was determined by Etest. MICs, except for colistin and tigecycline, were interpreted according to CLSI breakpoints [1]. EUCAST breakpoints were used for colistin susceptibility (http://www.eucast.org/clinical_breakpoints) and U.S. FDA breakpoints were used for tigecycline susceptibility [2].

Whole genome sequencing and comparative genomics

DNA of the *mcr-1*-harboring and carbapenemase-producing strain (KP2509) was extracted using Gentra Puregene Yeast/Bact kit (Qiagen) and subjected to Illumina Hiseq and PacBio SMRT sequencing as described previously [3]. A hybrid assembly using both Illumina and PacBio reads was performed using Unicycler 0.4.8 [4]. Genomes were annotated using the NCBI prokaryotic genome annotation pipeline [5]. The multi-locus sequence type (MLST) and capsular *wzi* allele were determined using

the *K. pneumoniae* MLST website (https://bigsdb.pasteur.fr/klebsiella), while the acquired resistance genes and plasmid replicons were determined respectively by Resfinder and PlasmidFinder online tools hosted on the Centre for Genomic Epidemiology website (genomicepidemiology.org) [6, 7]. CRISPR-Cas systems were identified using CRISPRCasFinder and CRISPRCasTyper [8, 9].

Construction of *mcr-1*-bearing recombinant plasmid

Plasmid vector pJET-TC (5,674 bps) was generated by cloning the tetracycline resistant gene (*tetA*) into a pJET1.2/blunt vector (Thermo Fisher Scientific). The *mcr-1* fragment in KP2509 was amplified by PCR with primers (*mcr-1*-promoter-F 5'-aagatacaaattataaatactctcaagtg-3' and *mcr-1*+1626-R 5'-tcagcggatgaatgcggt-3'), and the resulting PCR product was ligated into blunted BglII-digested pJET-TC. This *mcr-1*-bearing recombinant plasmid was designated as pJET-TC-MCR-1 (7,489 bps). We then transformed the pJET-TC-MCR-1 and pJET-TC plasmids into the *mcr-1* plasmid-cured strain PC-KP2509, and compared their stabilities in the common host. The transformants were passaged daily without additional antibiotics for 28 days. Colonies were inoculated onto tetracycline-containing and antibiotic-free LB agar plates, as bacteria losing the pJET-TC or the pJET-TC-MCR-1 plasmid will be susceptible to tetracycline. The number of colonies harbouring recombinant plasmids was calculated.

In addition, we compared the *mcr-1* gene copy numbers at logarithmic phase between KP2509 and pJET-TC-MCR-1-bearing PC-KP2509. A single colony was inoculated into Luria-Bertani broth and grown at 37°C in a shaking incubator (200 rpm) overnight. Refresh overnight cultures as mentioned above for 2.5 hours and then genomic DNA from two strains were extracted using Presto™ Mini gDNA Bacteria Kit (Geneaid). We used real-time quantitative PCR (qPCR) analysis of genomic DNA to determine *mcr-1* copy numbers relative to an internal control gene, *16 S*. Real-time qPCR was performed on Applied Biosystems 7500 Fast Real-Time PCR System (Thermo Fisher) using Fast SYBR Green Master Mix (Thermo Fisher).

SUPPLEMENTARY RESULTS

Whole genome sequencing of strain KP2509

Analysis of acquired antimicrobial resistance identified 21 antimicrobial resistance genes encoding resistance to β-lactams ($bla_{\text{TEM-1}}$, $bla_{\text{SHV-11}}$, $bla_{\text{SHV-12}}$, $bla_{\text{CTX-M-65}}$, $bla_{\text{KPC-2}}$ and $bla_{\text{OXA-48}}$), aminoglycosides [*aph(3'')-Ib*, *aph(6)-Id*, *aph(3')-Ia*, *aac(3)-IIe*, *aadA1* and *aadA2*], fluoroquinolones (*qnrS1*), phenicol (*cmlA1* and *catA2*), fosfomycin (*fosA*), tetracycline [*tet(D)*], sulphonamide (*sul3*), trimethoprim (*dfrA14*), colistin (*mcr-1*) and quaternary ammonium (*qacL*). In addition, examination of the sequences in the quinolone resistance-determining region (QRDR) in *gyrA*, *gyrB*, *parC* and *parE*

identified mutations encoding amino acid substitutions at Ser83-Ile and Asp87-Gly in *gyrA*, and Ser80-Ile in *ParC*, respectively.

Genomic characterization of plasmids in KP2509

The five plasmids in KP2509 belong to IncHI-FIB-N, IncFII-R, IncL, IncN, and ColRNAI incompatibility groups, respectively, and harbored a total of 19 antimicrobial resistance genes. Among them, bla_{KPC-2} is carried on plasmid pKP2509-KPC, which is 100,684 bp in length, and co-carries ESBL resistance genes $bla_{CTX-M-65}$ and bla_{SHV-12}, and chloramphenicol resistance gene *catA2* (Figure S1A). bla_{KPC-2} is located on a previously described NTEKPC-Ib element [10]. pKP2509-KPC carries two replicons of IncFII(pHN7A8) and IncR. IncF plasmid replicon sequence typing assigned pKP2509 as F33:A-:B-. Blast search of pKP2509-KPC against NCBI completed genomes and plasmid database showed that pKP2509-KPC had 90-96% query coverage and over 99% sequence identities with several plasmids isolated from *K. pneumoniae* in mainland China (*e.g.* pF1_1, CP026131; p12085-KPC, MN842292 and pA1750-KPC, MT108207). In comparison to a close related plasmid, pF1_1 (CP026131), from a clinical ST11 strain F1 in Fujian, China, pKP2509-KPC contains a ~56 kb deletion between the *tonB*-like and an oxidoreductase gene, encompassing the nearly entire *tra* operon. This deletion is presumed to be associated with the plasmid's inability to conjugate (Figure S1A).

Carbapenemase gene *bla*$_{OXA-48}$ was located on the IncL plasmid pKP2509-OXA, which is 63,769 bp in size. The global spread of OXA-48 carbapenemase, initially described in Turkey, has been primarily associated with the IncL pOXA48a-like plasmid [11]. The overall structure of pKP2509-OXA is similar to that of pOXA48, except that the *bla*$_{OXA-48}$ gene is located in Tn*1999.2* transposon in pKP2509-OXA, but in Tn*1999.1* in pOXA48. Blast analysis showed pKP2509-OXA is highly similar to several *bla*$_{OXA-48}$-harboring IncL plasmids found in Taiwan (e.g. pOXA48-L121, CP040031 and pOXA48-L117, CP040036) with 100% query coverage and overall > 99.9% nucleotide identity (Figure S1B).

The IncN plasmid pKP2509-4 is 49,502 bp in size and contains the plasmid-mediated quinolone resistance gene, *qnrS1*, and the trimethoprim-resistant gene, *dfrA14*. The fifth plasmid, pKP2509-5, is 10,060 bp in size, belongs to the ColE1 incompatibility group and it lacks antimicrobial resistance genes.

REFFERENCES

[1] Clinical and Laboratory Standards Institute. 2020. *Performance Standards for Antimicrobial Susceptibility Testing, 30th Edition.* Wayne, PA.

[2] U.S. Food and Drug Administration. Tygacil (tigecycline). https://www.accessdata.fda.gov/drugsatfda_docs/label/2013/021821s026s031lbl.pdf.

[3] Cheng YH, Huang TW, Juan CH, et al. Tigecycline-non-susceptible hypervirulent *Klebsiella pneumoniae* strains in Taiwan. J Antimicrob Chemother. 2020;75:309-317.

[4] Wick RR, Judd LM, Gorrie CL, et al. Unicycler: Resolving bacterial genome assemblies from short and long sequencing reads. PLoS Comput Biol. 2017;13:e1005595.

[5] Tatusova T, DiCuccio M, Badretdin A, et al. NCBI prokaryotic genome annotation pipeline. Nucleic Acids Res. 2016;44:6614-24.

[6] Bortolaia V, Kaas RS, Ruppe E, et al. ResFinder 4.0 for predictions of phenotypes from genotypes. J Antimicrob Chemother. 2020;75:3491-3500.

[7] Carattoli A, Zankari E, Garcia-Fernandez A, et al. *In silico* detection and typing of plasmids using PlasmidFinder and plasmid multilocus sequence typing. Antimicrob Agents Chemother. 2014;58:3895-903.

[8] Couvin D, Bernheim A, Toffano-Nioche C, T et al. CRISPRCasFinder, an update of CRISRFinder, includes a portable version, enhanced performance and integrates

search for Cas proteins. Nucleic Acids Res. 2018;46:W246-W251.

[9] Russel J, Pinilla-Redondo R, Mayo-Munoz D, et al. CRISPRCasTyper: automated identification, annotation, and classification of CRISPR-Cas loci. CRISPR J. 2020;3:462-469.

[10] Chen L, Mathema B, Chavda KD, et al. Carbapenemase-producing *Klebsiella pneumoniae*: molecular and genetic decoding. Trends Microbiol. 2014;22:686-96.

[11] Poirel L, Heritier C, Tolun V, et al. Emergence of oxacillinase-mediated resistance to imipenem in *Klebsiella pneumoniae*. Antimicrob Agents Chemother. 2004;48:15-22.

SUPPLEMENTARY FIGURE 1

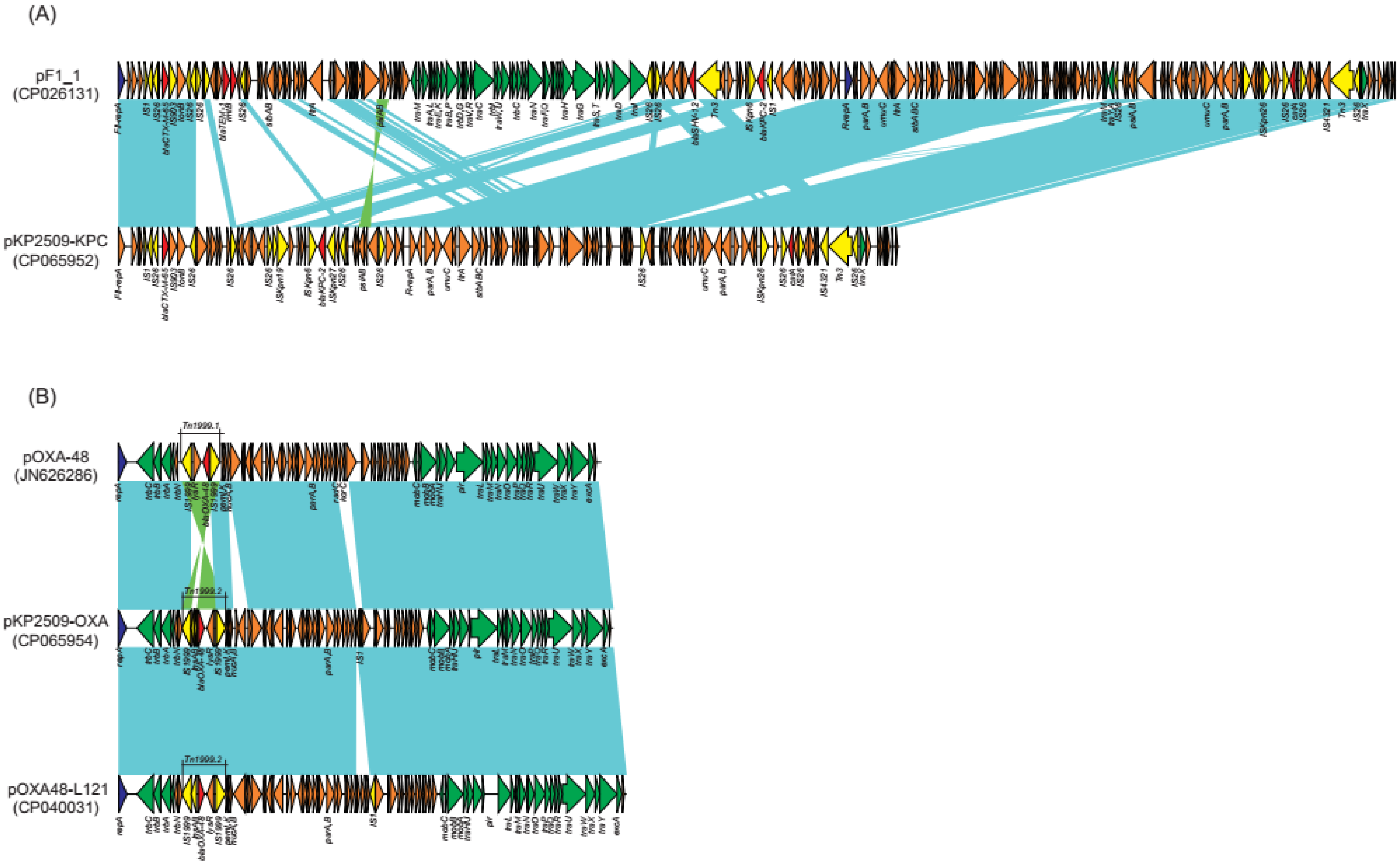

Figure S1 (A) Plasmid structures of *bla*$_{KPC-2}$-harboring IncFII-R plasmids. (B) Plasmid structures of *bla*$_{OXA-48}$-harboring IncL plasmids. Coloured arrows indicate open reading frames, with blue, yellow, green, red and orange representing replication genes, mobile elements, plasmid transfer genes, the antimicrobial resistance gene and plasmid backbone genes, respectively. Blue shading indicates shared regions of homology, while green shading indicates inversely displayed regions of homology.
